# Mobile Phone and Wearable Sensor-Based mHealth Approaches for Psychiatric Disorders and Symptoms: Systematic Review

**DOI:** 10.2196/mental.9819

**Published:** 2019-02-20

**Authors:** Jussi Seppälä, Ilaria De Vita, Timo Jämsä, Jouko Miettunen, Matti Isohanni, Katya Rubinstein, Yoram Feldman, Eva Grasa, Iluminada Corripio, Jesus Berdun, Enrico D'Amico, M-RESIST Group, Maria Bulgheroni

**Affiliations:** 1 Center for Life Course of Health Research University of Oulu Oulu Finland; 2 Department of Mental and Substance Use Services Eksote, Lappeenranta Finland; 3 Ab.Acus srl Milano Italy; 4 Research Unit of Medical Imaging, Physics and Technology University of Oulu Oulu Finland; 5 Medical Research Center Oulu Oulu University Hospital and University of Oulu Oulu Finland; 6 Department of Diagnostic Radiology Oulu University Hospital Oulu Finland; 7 The Gertner Institute for Epidemiology and Health Policy Research Tel Aviv Israel; 8 Department of Psychiatry Biomedical Research Institute Sant Pau (IIB-SANT PAU) Hospital Sant Pau Barcelona Spain; 9 Universitat Autònoma de Barcelona (UAB) Barcelona Spain; 10 CIBERSAM Madrid Spain; 11 Fundació TIC Salut Social Barcelona Spain; 12 m-RESIST Barcelona Spain

**Keywords:** sensors, mobile phone, m-RESIST, ecological momentary assessment, EMA, psychiatric disorder, schizophrenia

## Abstract

**Background:**

Mobile Therapeutic Attention for Patients with Treatment-Resistant Schizophrenia (m-RESIST) is an EU Horizon 2020-funded project aimed at designing and validating an innovative therapeutic program for treatment-resistant schizophrenia. The program exploits information from mobile phones and wearable sensors for behavioral tracking to support intervention administration.

**Objective:**

To systematically review original studies on sensor-based mHealth apps aimed at uncovering associations between sensor data and symptoms of psychiatric disorders in order to support the m-RESIST approach to assess effectiveness of behavioral monitoring in therapy.

**Methods:**

A systematic review of the English-language literature, according to the Preferred Reporting Items for Systematic Reviews and Meta-Analyses (PRISMA) guidelines, was performed through Scopus, PubMed, Web of Science, and the Cochrane Central Register of Controlled Trials databases. Studies published between September 1, 2009, and September 30, 2018, were selected. Boolean search operators with an iterative combination of search terms were applied.

**Results:**

Studies reporting quantitative information on data collected from mobile use and/or wearable sensors, and where that information was associated with clinical outcomes, were included. A total of 35 studies were identified; most of them investigated bipolar disorders, depression, depression symptoms, stress, and symptoms of stress, while only a few studies addressed persons with schizophrenia. The data from sensors were associated with symptoms of schizophrenia, bipolar disorders, and depression.

**Conclusions:**

Although the data from sensors demonstrated an association with the symptoms of schizophrenia, bipolar disorders, and depression, their usability in clinical settings to support therapeutic intervention is not yet fully assessed and needs to be scrutinized more thoroughly.

## Introduction

mHealth (ie, mobile health) is the intersection of electronic health and mobile devices for medicine and public health administration [1]. Many studies have actively exploited mHealth to provide questionnaires and qualitative feedback to facilitate treatment accessibility and participant retention or to monitor symptoms and treatment progress in a qualitative way. This is widely done using ecological momentary assessment (EMA) performed through e-diaries recording participants’ behavior. EMA collects self-report data through a variety of change-sensitive questionnaires [2-6]. However, self-monitoring has not always been shown to be a valid measurement of behavior. For example, a systematic review pointed out that electronic self-monitoring of mood among depression sufferers appeared to be a valid measure of mood in contrast to self-monitoring of mood among mania sufferers [7].

The rapid growth of smart-sensor integration in mobile phones and wearable devices has opened the prospect of increasing access to evidence-based mental health care. Mobile devices allow the collection of quantitative behavioral and functional markers in a transparent and unobtrusive way, providing an estimation of physiological and mental state [8-11]. A mobile phone-based approach may be valuable in gathering long-term objective data, aside from self-ratings, to predict changes in clinical states and to investigate causal inferences about state changes in patients (eg, those with affective disorders) [12].

In this review, the term *sensor-based data* includes the quantitative information supplied by the mobile phone and its embedded sensors. Information may range from acceleration to temperature and from light to pressure, but also from number of exchanged short message service (SMS) text messages to number of incoming and outgoing calls. Indeed, the variety of personal data, easily acquirable in this way, offers a unique opportunity to describe the person in terms of his or her lifestyle and behavior at the physical, cognitive, and environmental level [13,14].

Even if the evidence of association between sensor-based data and psychiatric disorder status and/or severity of psychiatric symptoms is limited and scattered [15-17], it is expected that appropriate management of these data may initiate a new trend in health care provision characterized by tailored and timely interventions [18].

Substantial treatment improvements have been achieved for several psychiatric disorders in the past decades. Nevertheless, the functional recovery of patients with schizophrenia is still low [19]. Treatment-resistant schizophrenia (TRS), especially, has a wide impact on the humanistic burden, which concerns patients and caregivers and involves several dimensions, such as quality of life, treatment side effects, caregiver burden, social impairment, suicide, violence, and healthy lifestyle [20]. Moreover, TRS patients show poor adherence to treatment-as-usual (TAU) intervention programs, which, in turn, cannot ensure continuity of assistance, immediacy of attention, tailored treatment, and caregivers’ integration [21]. In this context, the Mobile Therapeutic Attention for Patients with Treatment-Resistant Schizophrenia (m-RESIST) project [22] addresses patients with TRS by allowing caregivers and professionals to utilize mobile technology as part of the care process. These interventions determine a personalized flow of information based on a “Need 4 Help” scale and the stratification of patients depending on their risk level. m-RESIST is composed of three main parts: (1) a mobile phone connected to a smartwatch for patients and caregivers; (2) a Web-based dashboard for follow-up and monitoring by clinicians; and (3) a back-end system for managing data, interventions, and interactions between users [23].

The aim of this paper is to systematically review original studies on sensor-based data collection, targeting correlations between objective measurements of personal data and symptoms of psychiatric disorders to support the m-RESIST clinical approach. The main goal is to assess the perspective of integrated sensor-based mHealth interventions to deliver highly personalized mental care, monitoring the individual and his or her own modification along the way.

## Methods

### Overview

This systematic review has been performed according to the Preferred Reporting Items for Systematic Reviews and Meta-Analyses (PRISMA) guidelines [24]. Accordingly, strict eligibility criteria were applied in order to identify journal articles and reviews addressing the collection of sensor-based data in mental health and to investigate the association between sensor-based data and mental state. For a detailed description, see the PRISMA checklist in Multimedia Appendix 1.

### Eligibility Criteria

Eligibility criteria are listed in Textbox 1.

**Eligibility criteria of papers to be included in this review.**

- Types of participants: papers that studied participants with mental disorder diagnoses or symptoms of mental disorders (eg, depression, anxiety, sleep disorders, psychotic disorders, stress, and panic disorders) were included; papers that studied participants without mental disorder diagnoses, but that analyzed participants to identify mental disorders or symptoms (eg, depression, anxiety, sleep disorders, and stress) were also included.
- Types of methods: studies reporting transparent and unobtrusive monitoring using commercially available wearable sensors (eg, wristbands, bracelets, smartwatches, and mobile phones) were included. Studies describing Internet-based interventions, interactive voice-response technologies, and self-reporting interventions based on questionnaires without a sensor-based mobile app component were excluded. Furthermore, studies using obtrusive monitoring devices (eg, chest band and helmets) were also excluded.
- Types of outcomes: studies reporting results associating mental health status and sensor-based data were included. Papers providing a description of the mobile app, but no statistical outcomes, were excluded.
- Language and time frame: English-language full-text articles, reviews, and conference abstracts were included in the review. Considering the trend of technology evolution, papers published between January 1, 2009, and September 30, 2018, were included.

### Information Sources, Search Strategy, and Study Selection

The search for papers was performed using the following electronic databases: Scopus, PubMed, Web of Science, and the Cochrane Central Register of Controlled Trials. The following combinations of search terms were used: (“mental health” OR “mental disorder” OR depression OR anxiety OR psychosis OR schizophrenia OR “treatment resistant schizophrenia” OR bipolar OR insomnia OR stress) AND (mobile OR smartphone) AND (monitor OR sensing OR sensor).

Results of the search were made available in Excel files and included the title, authors, source, date, and abstract for study selection. Duplicated studies were removed before starting the selection. An eligibility check was performed on the title, keywords, and abstract of each study. Full-text copies of all potentially relevant papers, or papers where there was insufficient information in the abstract to determine eligibility, were obtained.

Study selection, according to the eligibility criteria described in Textbox 1, was performed independently by two reviewers: one with a clinical background and one with technological background. There were no cases of disagreements between the two reviewers.

The extracted information consisted of the following: (1) sensors that were used; (2) computed parameters; (3) participants (ie, number and state of health); and (4) relation to clinical outcomes.

## Results

**Figure 1:**
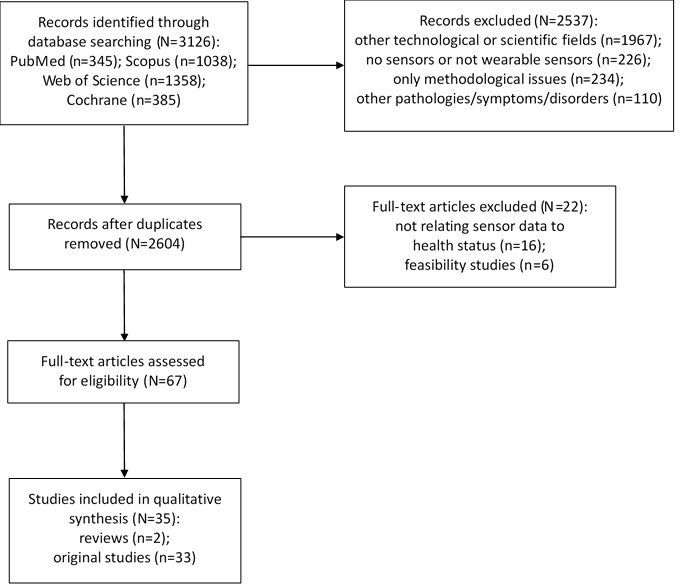
Flowchart of study selection process.

As summarized in Figure 1, a total of 345 unique records were found from PubMed, 1038 from Scopus, 1358 from Web of Science, and 385 from the Cochrane Central Register of Controlled Trials, for a total number of 3126 hits. In all, 522 duplicates among the four databases were identified and removed.

A total of 1967 additional records were excluded because they reported on other technologies and/or other scientific fields. Another 226 were excluded because they did not report on suitable wearable sensors or did not report on sensors at all. An additional 234 were excluded because they described mainly methodological issues (eg, protocols of analyses, mobile phone-based monitoring, and treatment apps) without suitable testing of subjects. Another 110 were excluded because they addressed pathologies, symptoms, and disorders outside of the mental health domain.

Altogether, 67 full-text papers were read; of these, 16 were excluded because they did not relate sensor data to health status assessment [25-40], while another six were feasibility studies with no relation to health status assessment [41-46].

In all, 35 articles were included in this review; two of them were complete reviews. One complete review addressed the association between a collection of behavioral features from mobile phones and wearable sensors with depressive mood symptoms in patients with affective disorders [47]. The other complete review addressed the use of digital health technology in the wider domain of serious mental illness [48]. Association of depressive mood symptoms with social behavior assessed through phone usage, physical activity measured through accelerometer and gyroscope, location measured by GPS, and overall device usage was not consistent across all studies [47,48]. The other 33 original papers are summarized in Table 1 [49-81].

**Table 1:** Summary of original papers.

Source	Sample description	Collected data	Related clinical measures	Results
Ben-Zeev et al [49]	47 healthy subjects	GPS, accelerometer, gyroscope, microphone, and light sensor	PHQ-9^a^, PSS^b^, and revised UCLA^c^ loneliness scale	Speech duration, sleep duration, and geospatial activity relate to PHQ-9; kinesthetic activity relates to UCLA loneliness scale.
Osmani V et al [50]	9 subjects with bipolar disorders	Accelerometer and gyroscope	HAMD^d^ and YMRS^e^	Psychiatric assessment scores relate to physical activity level at specific time intervals of the day.
Chow P et al [51]	72 healthy subjects	GPS	SIAS^f^ and DASS-21^g^	Social anxiety and depression relate to time spent at home in specific time intervals of the day.
Boukhechba et al [52]	54 healthy subjects	GPS, phone calls, and messages	SIAS	Social anxiety relates to limited social life and reduced mobility.
Staples et al [53]	17 subjects with schizophrenia	Accelerometer and gyroscope	PSQI^h^	Moderate correlation between sleep estimate and PSQI.
Sano et al [54]	66 healthy subjects	Accelerometer, gyroscope, skin temperature, skin conductance, phone calls, messages, and screen on/off	PSQI, Big Five Inventory Personality Test, MEQ^i^, PSS, and MCS for mental health^j^	PSQI and stress relate to phone usage.
Sano et al [55]	18 healthy subjects	GPS, accelerometer, gyroscope, skin conductance, phone calls, messages, and screen on/off	PSS, PSQI, and Big Five Inventory Personality Test	Stress relates to phone usage and physical activities at specific time intervals of the day.
Stutz et al [56]	15 healthy subjects	Accelerometer, gyroscope, light, app usage, and screen on/off	PSS	PSS relates mainly to phone usage.
Difrancesco et al [57]	7 subjects with schizophrenia	GPS	Birchwood’s Social Functioning Scale	Locations detected through GPS relate well to the activities identified in the social functioning scale.
Osmani V [58]	12 subjects with bipolar disorders	GPS, accelerometer, gyroscope, and microphone	Mental scale (not specifically defined)	Physical activity and voice features relate to the patient’s state.
Renn B et al [59]	600 subjects with depression	GPS	PHQ-2^k^	Limited association between mobility and depressive symptoms rating.
Mehrotra et all [60]	25 healthy subjects	Phone notification management (eg, clicks, decision, and response time), phone calls, and app usage	PHQ-8^l^	Moderate correlation between depression state and notification management as well as phone and app usage in a 14-day period; limited correlation on shorter periods of time.
Grunerbl et al [61]	10 subjects with bipolar disorders	GPS, accelerometer, gyroscope, microphone, and phone calls	HAMD and YMRS	Good relationship between sensor data and the patient’s state.
Saeb et al [62]	28 healthy subjects	GPS and phone usage	PHQ-9	Good relationship between phone usage (ie, calls and duration) and depression symptoms as well as GPS processed data and depression symptoms.
Guidi et al [63]	1 patient with bipolar disorder	Microphone	QID^m^ and YMRS	No clear relationship between voice features and clinical assessment.
Beiwinkel et al [64]	13 subjects with bipolar disorders	GPS, phone calls, and messages	HAMD and YMRS	Phone usage relates positively to depression state while activity relates negatively to manic symptoms.
Wahle et al [65]	126 healthy subjects	GPS, accelerometer, and phone usage	PHQ-9	Depression symptoms relate to mobile phone extracted features.
Shin et al [66]	61 patients with schizophrenia, DSM-IV^n^	Fitbit (ie, activity tracker)	PANSS^o^	Psychiatric symptoms relate to lower activity level.
Palmius et al [67]	29 subjects with bipolar disorders and 20 controls	GPS	QID	Location recordings relate to depressive episodes.
Abrantes et al [68]	20 subjects with alcohol use disorders	Fitbit (ie, activity tracker)	PHQ-9	Physical activity correlates with reduction in the level of depression and anxiety.
Saeb et al [69]	48 healthy subjects	GPS	PHQ-9	GPS correlates with depression differently on weekdays and weekends.
Place et al [70]	73 subjects with at least one symptom of depression	GPS, accelerometer, gyroscope, phone calls, messages, microphone, and screen on/off	Semi-structured clinical interview	Physical activity and phone usage relate to depression symptoms.
Saeb et all [71]	206 healthy subjects	GPS, accelerometer, gyroscope (Android activity-recognition API^p^), light sensor, microphone, screen on/off, phone calls, and messages	PHQ-9 and GAD-7^q^	No consistent relationship between GPS-based semantic location and depression or anxiety.
Faurholt-Jepsen et al [72]	61 subjects with bipolar disorders	Phone calls and messages	HAMD and YMRS	Significant correlation between depressive and manic symptoms and phone usage.
Sabatelli et al [73]	7 subjects with bipolar disorders	Wi-Fi-based position	HAMD and YMRS	Weak negative correlation between staying in clinics and self-reported state.
Rabbi et al [74]	8 healthy subjects (elders)	Accelerometer, gyroscope, barometer, and microphone	Friendship Scale, SF-36^r^, CES-D^s^, and YPAS^t^	No clear relationship between sensor data and administered assessment scales.
Doryab et al [75]	3 healthy subjects	GPS, accelerometer, gyroscope, microphone, and light sensor	CES-D	Correlation between depression scales and sensor data.
Farhan et al [76]	60 healthy subjects	GPS, accelerometer, gyroscope, microphone, phone lock and unlock, light sensor, and phone call duration	PHQ-9	Correlation between PHQ-9 scores and all the sensor data is pointed out.
Canzian et al [77]	28 healthy subjects	GPS	PHQ-8	Significant correlation between mobility patterns and depressive mood.
Zulueta et al [78]	16 subjects with bipolar disorders	Phone keyboard usage	HAMD and YMRS	Accelerometer activity while typing, number of exchanged messages, and typing errors correlate with depression and mania scores.
Sano et al [79]	201 healthy subjects	Skin conductance, skin temperature, accelerometer, ambient light, GPS, phone calls, messages, app usage, and phone lock and unlock	PSS and MCS^u^	Skin conductance relates to MCS, skin temperature, and phone usage timing and duration; GPS relates both to PSS and MCS.
Tron et al [80]	25 subjects with schizophrenia, DSM-IV	Accelerometer, light, temperature	PANSS	Physical activity relates to PANSS.
Cella et al [81]	30 subjects with schizophrenia, DSM-IV, and 25 controls	Accelerometer, skin conductance, heart rate variability, and interbeat intervals	PANSS	Interbeat intervals negatively correlate with positive symptoms; movement negatively correlates with negative symptoms.

^a^PHQ-9: Patient Health Questionnaire-9.

^b^PSS: Perceived Stress Scale.

^c^UCLA: University of California, Los Angeles.

^d^HAMD: Hamilton Depression Rating Scale.

^e^YMRS: Young Mania Rating Scale.

^f^SIAS: Social Interaction Anxiety Scale.

^g^DASS-21: Depression, Anxiety, and Stress Scale.

^h^PSQI: Pittsburgh Sleep Quality Index.

^i^MEQ: Horne-Ostberg Morningness-Eveningness Questionnaire.

^j^MCS for mental health: Short Form-12 Physical and Mental Health Composite Scale.

^k^PHQ-2: Patient Health Questionnaire-2.

^l^PHQ-8: Patient Health Questionnaire-8.

^m^QID: Quick Inventory of Depression.

^n^DSM-IV: Diagnostic and Statistical Manual of Mental Disorders, Fourth Edition.

^o^PANSS: Positive and Negative Syndrome Scale.

^p^API: application programming interface.

^q^GAD-7: General Anxiety Disorder questionnaire.

^r^SF-36: Short Form-36 Health Survey.

^s^CES-D: Center for Epidemiologic Studies-Depression scale.

^t^YPAS: Yale Physical Activity Survey.

^u^MCS: Mental Component Summary.

Only five studies addressing schizophrenia were included in this review [53,57,66,80,81]. None of them included patients with treatment-resistant schizophrenia. Early studies by Ben-Zeev et al [34,49] analyzed patients’ location, activity, and speech, but did not associate sensor data to the severity of symptoms. Difrancesco et al [57] implemented a time-based method and a density-based method to identify the geolocations visited by 5 schizophrenic patients, detecting patients’ out-of-home activities with moderate recall. Staples et al [53] investigated sleep estimation of 17 patients by comparing the Pittsburgh Sleep Quality Index (PSQI), EMAs, and accelerometer data, but did not address the severity of symptoms. Psychiatric symptoms evaluated by the Positive and Negative Syndrome Scale (PANSS) among those with schizophrenia were related to lower activity level [66,80,81], while interbeat intervals correlated negatively with positive symptoms [81].

Nine studies were conducted among bipolar disorder patients [50,58,61,63,64,67,72,73,78]. Among those with bipolar disorder, physical activity was related to psychiatric assessment scores [50,58,61], but the association of voice features and patients’ psychiatric evaluation was incongruent [58,63]. A correlation between depressive and manic symptoms and phone usage was also detected [64,72]. Location recordings correlated with depressive symptoms and a weak negative association between staying in clinics and self-reported state was found [67,73]. Typing features (ie, interkey delay, backspace ratio, and autocorrect rate) were positively related to depression and mania [78].

Most of the other included studies referred to depression [59,70] or symptoms of depression and anxiety in healthy subjects [49,51,52,54-56,60,62,65,68,69,71,74-77,79]. In one study, a limited association was found between mobility and ratings of depressive symptoms [59], while physical activity and phone usage were related to depressive symptoms in another [70]. In healthy subjects with symptoms of depression and anxiety, several data such as speech, sleep duration, mobility, and phone usage were related to severity of symptoms [49,51,52,54-56,60,62,65,74-77], while GPS-based semantic location did not correlate with depression or anxiety [71].

## Discussion

### Principal Findings

The data from sensors were associated with symptoms of schizophrenia, bipolar disorder, and depression. This may have the potential to change the nature of identification, follow-up, and treatment of mental disorders. Early identification of behavioral markers of psychiatric disorders may allow health care providers to react early to patients’ needs and deliver personalized dynamic treatment.

This systematic review uncovered a broad investigation, but still limited use, of data coming from mobile phones and wearable sensors to support therapeutic intervention for psychiatric disorders or for psychiatric symptoms. This review showed a high variability in participant selection criteria, investigation protocols, and data processing techniques, which limits the generalizability of the identified associations between sensor-based data and clinical assessment. This was also seen in three recent studies in the area of passive sensing in the mental health domain and the wider health care domain [13,47,82]. The available studies in this review often had methodological limitations (eg, small sample size, variations in the number of observations or monitoring duration, lack of randomized control group, and heterogeneity of methods).

In addition, there were issues related to usability of sensors and acceptance by patients; risks (eg, they may increase psychotic experiences and fears), feasibility (eg, psychiatric patients may have cognitive and economic limitations), risk-benefit ratio, costs, and health economics were not widely investigated. Also, potential biases in measurements due to the individual usage of the devices were only marginally addressed in most of the selected papers; for example, practical mobile phone use modalities (eg, only at work or at home) or reliability of wearable sensors (eg, a tight or loose smartwatch bracelet).

On the other hand, current psychiatric evaluation is strongly limited by assessment through scales on the day of the visit with the clinician and not necessary during a crisis (eg, “bad day” or relapse situation); it does not appropriately reflect the subjective experience of the patient nor the impact of the treatment in real life. The benefits of sensor-based data information may also be useful among those with TRS, as they show poor adherence to TAU programs of intervention; TAU intervention programs cannot ensure continuity of assistance, immediacy of attention, tailored treatment, and caregiver integration [21].

The data collected from sensors is expected to strongly contribute to behavioral monitoring and mental status assessment over time on an individual basis in a transparent way. Within an intraperson investigation, the data may be used as a trigger to personalized interventions facilitating the implementation of remote psychiatric therapeutic programs. It is expected that the long-term analysis of sensor-based data, building on a personal baseline and assessing individual modifications, may play a key role in clinical applications [14]. To realize this, all aspects of mobile phone sensor technology should be thoroughly investigated. Studies using rigorous methodology are needed to investigate the beneficial as well as the harmful effects of extracting behavioral markers of psychiatric disorders or symptoms from sensor-based data.

### m-RESIST Project Contribution

Building on the results of this review, m-RESIST set up a framework to create a clinical decision support system (CDSS) based on a mobile therapeutic intervention for schizophrenic patients. The CDSS is designed to provide the users with necessary information to support health-related and clinical decision-making. The system utilizes available data sources in order to assess the patient’s condition using decision algorithms and, as a result, classify the clinical condition in order to provide clinical and lifestyle recommendations. The CDSS starts with a training period of two weeks, during which sensor-based data are collected, without activation of further system actions, in order to assess the patient’s baseline. Once trained, the system monitors the changes against the baseline. The functionality of the CDSS is based on the workflows developed by expert clinicians, reflecting the process of interaction between the system and its users in order to establish novel health care pathways. The CDSS activation is triggered by an event (ie, change in the baseline value) that is interpreted in a context of additional information that exists regarding a specific patient (ie, records in the patient’s file and information regarding attendance of scheduled visits) and a series of predefined conditions and actions [83].

The features supplied by sensor data that are used to trigger the CDSS are as follows: app number and duration of incoming, outgoing, and missed calls; number of incoming and outgoing SMS text messages by mobile phone; amount of time spent at home and in other places, measured by GPS data; and amount of time sleeping measured by physiological heart rate [83].

### Conclusions

The data from sensors are associated with symptoms of schizophrenia, bipolar disorder, and depression, but their usability in clinical practice needs to be scrutinized more thoroughly. m-RESIST aims to support intervention administration by sensor-based data in TRS. m-RESIST also plans to go a step further in remote therapy management of TRS by implementing a CDSS to correlate clinical information and sensor-based data. In m-RESIST, a mental status evaluation based on the most common perceptions and risk behaviors of patients with schizophrenia has been developed, together with the usual clinical scales. A pilot study has been carried out and its results are under analysis.

## Appendix

Multimedia Appendix 1 PRISMA checklist.

## Abbreviations

API: application programming interface
CDSS: clinical decision support system
CERCA: Centres de Recerca de Catalunya
CES-D: Center for Epidemiologic Studies-Depression scale
DASS-21: Depression, Anxiety, and Stress Scale
DSM-IV: Diagnostic and Statistical Manual of Mental Disorders, Fourth Edition
FEDER: Fonds Européen de Développement Économique et Régional
GAD-7: General Anxiety Disorder questionnaire
EMA: ecological momentary assessment
HAMD: Hamilton Depression Rating Scale
MCS: Mental Component Summary
MCS for mental health: Short Form-12 Physical and Mental Health Composite Scale
MEQ: Horne-Ostberg Morningness-Eveningness Questionnaire
m-RESIST: Mobile Therapeutic Attention for Patients with Treatment-Resistant Schizophrenia
PANSS: Positive and Negative Syndrome Scale
PHQ-2: Patient Health Questionnaire-2
PHQ-8: Patient Health Questionnaire-8
PHQ-9: Patient Health Questionnaire-9
PRISMA: Preferred Reporting Items for Systematic Reviews and Meta-Analyses
PSQI: Pittsburgh Sleep Quality Index
PSS: Perceived Stress Scale
QID: Quick Inventory of Depression
SF-36: Short Form-36 Health Survey
SIAS: Social Interaction Anxiety Scale
SMS: short message service
TAU: treatment-as-usual
TRS: treatment-resistant schizophrenia
UCLA: University of California, Los Angeles
YMRS: Young Mania Rating Scale
YPAS: Yale Physical Activity Survey
